# Comparison of the proximal and distal approaches for axillary vein catheterization under ultrasound guidance (PANDA) in cardiac surgery patients susceptible to bleeding: a randomized controlled trial

**DOI:** 10.1186/s13613-020-00703-6

**Published:** 2020-07-08

**Authors:** Ying Su, Jun-yi Hou, Guo-guang Ma, Guang-wei Hao, Jing-chao Luo, Shen-ji Yu, Kai Liu, Ji-li Zheng, Yan Xue, Zhe Luo, Guo-wei Tu

**Affiliations:** 1 Department of Critical Care Medicine, Zhongshan Hospital, Fudan University, No. 180 Fenglin Road, Xuhui District, Shanghai, 200032 China; 2 Department of Nursing, Zhongshan Hospital, Fudan University, No. 180 Fenglin Road, Xuhui District, Shanghai, 200032 China; 3 Department of Critical Care Medicine, Xiamen Branch, Zhongshan Hospital, Fudan University, No. 668 Jinghu Road, Huli District, Xiamen, 361015 China

**Keywords:** Axillary vein, Subclavian vein, Central venous cannulation, Central venous access, Longitudinal axis

## Abstract

**Background:**

The present study aimed at comparing the success rate and safety of proximal versus distal approach for ultrasound (US)-guided axillary vein catheterization (AVC) in cardiac surgery patients susceptible to bleeding.

**Methods:**

In this single-center randomized controlled trial, cardiac surgery patients susceptible to bleeding and requiring AVC were randomized to either the proximal or distal approach group for US-guided AVC. Patients susceptible to bleeding were defined as those who received oral antiplatelet drugs or anticoagulants for at least 3 days. Success rate, catheterization time, number of attempts, and mechanical complications within 24 h were recorded for each procedure.

**Results:**

A total of 198 patients underwent randomization: 99 patients each to the proximal and distal groups. The proximal group had the higher first puncture success rate (75.8% vs. 51.5%, *p *< 0.001) and site success rate (93.9% vs. 83.8%, *p *= 0.04) than the distal group. However, the overall success rates between the two groups were similar (99.0% vs. 99.0%; *p *= 1.00). Moreover, the proximal group had fewer average number of attempts (*p *< 0.01), less access time (*p *< 0.001), and less successful cannulation time (*p *< 0.001). There was no significant difference in complications between the two groups, such as major bleeding, minor bleeding, arterial puncture, pneumothorax, nerve injuries, and catheter misplacements.

**Conclusions:**

For cardiac surgery patients susceptible to bleeding, both proximal and distal approaches for US-guided AVC can be considered as feasible and safe methods of central venous cannulation. In terms of the first puncture success rate and cannulation time, the proximal approach is superior to the distal approach. *Trial registration* Clinicaltrials.gov, NCT03395691. Registered January 10, 2018, https://clinicaltrials.gov/ct2/show/NCT03395691?cond=NCT03395691&draw=1&rank=1.

## Background

Central venous catheterization (CVC) is a frequent procedure in cardiac surgery patients [1]. Subclavian vein catheterization (SVC) with less risk of infection and thrombosis and more comfort was the preferred option during the postoperative period [2, 3]. However, when implementing SVC, the rate of mechanical complications such as arterial puncture, hemothorax, and hematoma was low but persistent even when performed by experts [4]. Ultrasound (US) has become a widely accepted procedure to guide safe and accurate CVC [5, 6]. As the subclavian vein is not easily visualized by US because of the clavicle, the axillary vein in the infraclavicular area is an alternative choice [7]. US-guided axillary vein catheterization (AVC) showed higher success rate and fewer complications than the landmark method [8]. Two puncture approaches (proximal and distal) are usually used for AVC in clinical settings [9–12]. The proximal infraclavicular axillary vein is a direct continuation of the subclavian vein. The associated anatomy is simple as the proximal vein is straight and thick in the longitudinal axis view, which favors successful puncture [13, 14]. The distal axillary vein also has anatomical advantages for safe cannulation. The distal axillary vein lies further away from the artery and chest wall, and the overlap between the distal axillary vein and the artery increases laterally [9]. To date, there is no specific recommendation of using proximal or distal puncture approaches for US-guided AVC due to limited evidence. Only one study has compared the two puncture approaches in a randomized controlled trial, in which the success rates of distal and proximal approaches were similar in generally critically ill patients [15].

Medication-induced disordered hemostasis is common in cardiac surgery patients as antiplatelet drugs or anticoagulants are frequently used for preventing thrombosis [16]. The use of these drugs is associated with an increased risk of bleeding. Any invasive procedures including CVC might further put these patients at an additional risk of bleeding [17, 18].

To date, no study has compared the two above-mentioned puncture approaches in cardiac surgery patients susceptible to bleeding. The present study aimed to compare the success rate and safety of proximal and distal approaches for US-guided AVC in cardiac surgery patients susceptible to bleeding.

## Methods

### Trial design

The PANDA (comparison of the proximal and distal approaches of axillary vein catheterization under ultrasound guidance) trial was a single-center prospective randomized controlled trial. The protocol of the PANDA study was approved by the Ethics Committee of Zhongshan Hospital, Fudan University (Shanghai, China, IRB No. B2017-140) and registered at ClinicalTrials.gov (ID: NCT03395691). Written informed consent was obtained from legal representatives in accordance with the Declaration of Helsinki.

### Participants

Cardiac surgery patients of age > 18 years and requiring AVC for any clinical reason were screened. Patients susceptible to bleeding were defined as those who received oral antiplatelet drugs and/or anticoagulants for at least 3 days.

### Exclusion criteria

Patients meeting one of the following criteria were excluded: (1) the proximal and/or distal axillary vein was not clearly visualized or potentially unavailable for catheterization; (2) did not receive or had not received oral antiplatelet drugs and/or anticoagulants for less than 3 days; (3) already had presence of subclavian or axillary vein catheter; (4) required an emergency AVC; (5) had fracture of the ipsilateral clavicle or anterior proximal ribs; (6) had subclavian and/or axillary vein thrombosis; and (7) had local infection of the puncture area.

### Randomization

Patients were randomized to the proximal approach (PA) or distal approach (DA) group in a 1:1 ratio by using a computerized system. The allocation process was intensively managed by an allocation group using sequentially numbered containers, and the allocation result was concealed until it was implemented. When a patient was eligible, the investigator informed the allocation group to allocate the patient to the intervention group: PA or DA group. Because of feasibility issues, operators were not blinded to the assignment.

### Interventions

All procedures were performed in the Cardiac Surgery Intensive Care Unit (*n* = 39 beds) by five physicians with 3 to 18 months of experience in US-guided AVC. All operators underwent a protocolized training program to minimize variations in procedures before the initiation of this study [19]. A Philips CX50 system (Philips Healthcare, Eindhoven, Netherlands) equipped with a 3–12 MHz, linear array probe in the vascular mode was used for all CVCs. An 8 Fr two-lumen central venous catheter (Arrow International Inc., Reading, PA, USA) was chosen.

**Fig. 1:**
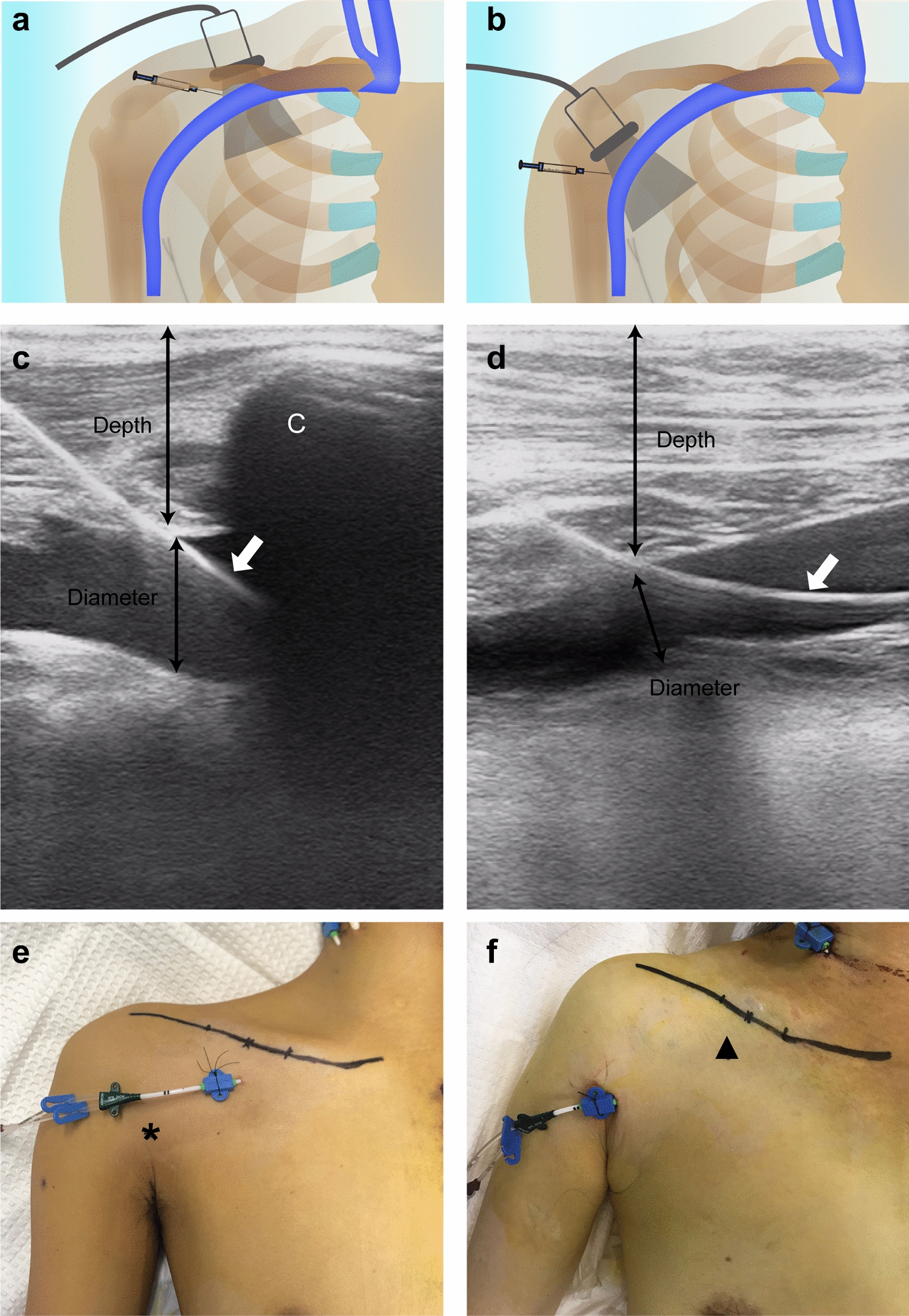
Illustration of proximal axillary (left) and distal axillary (right) approaches. **a**, **b** Diagrammatic drawings of the anatomical views of proximal (**a**) and distal (**b**) approaches for ultrasound-guided axillary venous catheterization. **c**, **d** Ultrasound visualization of the guide wire in the proximal (**c**) and distal (**d**) axillary veins. **e**, **f** catheter in place secured to the skin in proximal (**e**) and distal (**f**) approaches. The inferior margins of the clavicle are drawn in **e** and **f**, where the mid-clavicular point and the medial and lateral one-third of the clavicle are marked. White arrows show the guide wire. Black asterisk indicates the anticipated skin puncture site of the distal approach and black triangle indicates the anticipated skin puncture site of the proximal approach. C, clavicle; depth, the distance between skin surface and the anterior wall of vein; diameter, anteroposterior diameter of the vein

The proximal approach refers to the mid-infraclavicular axillary vein access (often termed as US-guided SVC in previous literature), in which the entry of the needle is close to the mid-clavicle (Fig. 1a). The distal approach refers to the more distal axillary vein access, in which the entry of the needle is distant from the artery and pleural cavity in the axilla (Fig. 1b). The patients were placed in the supine position with arms in the neutral position. A preoperative scanning was conducted to examine the axillary vein and the surrounding structures in the long-axis and short-axis view. Color Doppler imaging and flow measurements were used to confirm patency of the axillary vein. Lung sliding was examined to provide a comparison for post-procedure assessment. The operating field including the neck area was prepared with iodine disinfectant. The probe was wrapped in a sterile sleeve, with a sterile gel applied outside and inside the sleeve. The longitudinal axis/in-plane approach was used in which the target vein and the entire needle including the tip were simultaneously visualized during catheterization. The operators held the probe with the left (nondominant) hand and the needle with the right (dominant) hand. The probe was gently placed on the skin with the least amount of pressure to obtain the optimal image. The skin and subcutaneous tissues were infiltrated with lignocaine before venipuncture. When introducing the needle, the progression of the needle into the vein was visualized in real time. Although the US beam and needle advances could not be clearly viewed, the needle could be aligned slightly to visualize the needle tip clearly. The needle tip in the US image was manifested as a moving bright spot associated with distortion of the surrounding tissues. The transducer could be slid or tilted to follow the needle tip, if necessary. When the needle tip punctured the target vein successfully, blood was freely pulled into a syringe. The wire was then advanced carefully. The wire position was confirmed by the longitudinal axis image before vessel dilation. The length of catheter insertion was at the discretion of operators. Generally, a 14-cm to 16-cm catheter was used for the proximal approach and an 18-cm to 20-cm catheter was used for the distal approach according to the patient’s features. If the first two attempts of venipuncture failed, the procedure was converted to the other approach in the next two attempts. When both approaches (four attempts) failed, another site (the other axillary or the internal jugular vein) was chosen for cannulation by an experienced operator (G.W.T); this was regarded as failure. After successful catheterization, the presence of complications was assessed by US. The depth of the axillary vein (the distance between skin surface and the anterior wall of the vein) and the vessel diameters were measured in the longitudinal axis image by using software of the US machine (Fig. 1c, d). At least one chest X-ray or CT scan was performed within the following 24 h.

### Outcomes

The primary outcome was the first puncture success rate defined as the number of successful catheterization in the first attempt. The secondary outcomes were the site success rate (defined as the number of successful cannulation within the first two attempts), overall success rate [defined as the number of successful cannulation in the targeted axillary vein within four attempts (the first two attempts using the randomized approach and the third and fourth attempts using the nonrandomized approach)], access time (defined as the time between penetration of skin and aspiration of venous blood into the syringe), time to successful catheterization (defined as the time from skin puncture until the completion of catheter insertion), number of attempts (one attempt was defined as the passage of the skin with the needle and one venipuncture), and mechanical complications occurring within the 24-h follow-up period (major bleeding, minor bleeding, arterial puncture, pneumothorax, nerve injuries, and catheter misplacement). Major bleeding was defined as a decrease in hemoglobin to > 1.6 g/dL or a need for transfusion or hemodynamic instability due to bleeding or hemothorax after the procedure. Minor bleeding was defined as the development of swelling > 2 cm around the skin puncture site under ultrasonography imaging after 5 min of manual compression. Catheter misplacement was defined as placement of the catheter’s tip outside the right atrium or the superior vena cava. The number of attempts, access time, insertion time, and immediate complications were recorded by a designated investigator. A physician blinded to the study data read all chest X-ray/CT scans to determine mechanical complications such as hemothorax, pneumothorax, and catheter misplacement.

### Sample size calculation

The expected first puncture success rate in the DA group ranged from 67% to 76% based on the literature [10, 15, 20]. Considering the low experience of operators, the anticipated first puncture success rate was set as 67%. Assuming a difference of not less than 20% in the proportion of the first puncture success rate between the groups and a 10% attrition rate, we estimated that 198 patients (99 per group) were required to provide 90% power at a two-sided alpha level of 0.05.

### Statistical analysis

All analyses were performed by the intention-to-treat method. Categorical variables were expressed as numbers and percentages. For continuous variables, the normality of distribution was evaluated using the Kolmogorov–Smirnov test. Continuous variables were expressed as mean and standard deviations (SD) or median and interquartile ranges (IQR). Categorical variables were compared using the Chi-square test or Fisher’s exact test, and continuous variables were compared using Student’s *t* test or the Mann–Whitney *U* test as appropriate. Statistical analyses were performed with SPSS 13.0 (IBM Inc., Armonk, NY, USA). Two-sided *p* values of less than 0.05 were considered statistically significant.

## Results

**Fig. 2:**
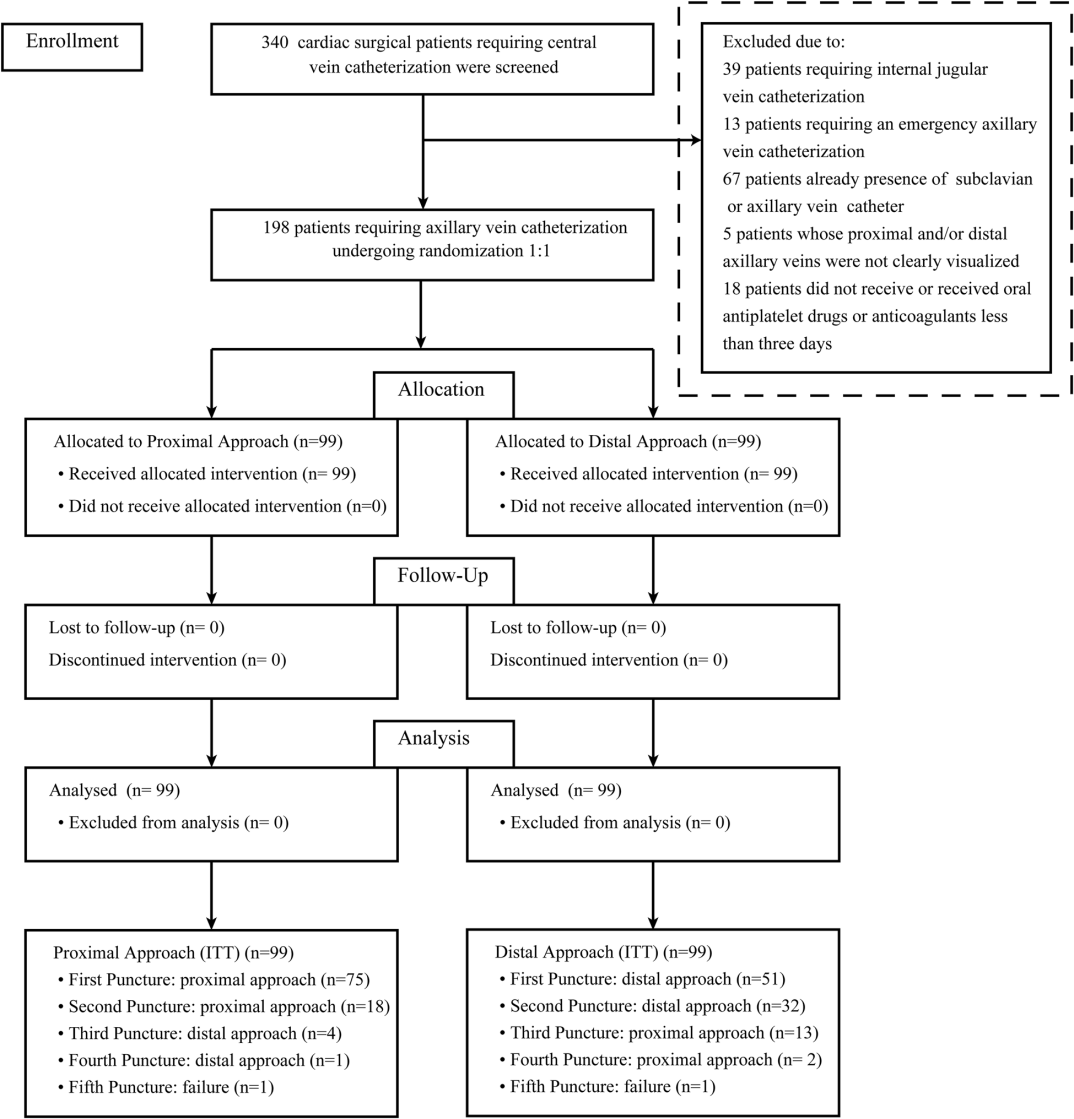
CONSORT flow diagram. ITT, intention-to-treat

From March 2018 to August 2019, a total of 340 patients were screened for inclusion, of whom 198 underwent randomization (99 in the PA group and 99 in the DA group). No randomized patients were excluded from the analysis (Fig. 2). There was no significant difference in baseline characteristics between the PA and DA groups (Table 1). In addition, major clinical parameters before venipuncture did not differ between the two groups (Table 2). The depth of the axillary vein in the PA group was smaller than that in the DA group (1.96 ± 0.51 cm vs. 2.45 ± 0.57 cm; *p *< 0.001), while the vessel diameter in the PA group was larger than that in the DA group (0.92 cm vs. 0.75 cm, IQR: 0.77–1.10 and 0.65–0.87, respectively; *p *< 0.001).

**Table 1:** Baseline clinical parameters of the study population

	Proximal group (*n* = 99)	Distal group (*n* = 99)	*p* value
Gender (male), *n* (%)	72 (72.7)	60 (60.6)	0.10
Age (years)	62 (53, 69)	60 (47, 68)	0.29
Body mass index (kg/m^2^)	24 ± 4.8	23.4 ± 4.0	0.34
Type of surgery, *n* (%)			
CABG only	17 (17.2)	12 (12.1)	0.42
Valve only	26 (26.3)	37 (37.4)	0.13
CABG and valve	10 (10.1)	8 (8.1)	0.81
Aortic surgery	31 (31.3)	28 (28.3)	0.77
Other cardiac surgery	15 (15.2)	14 (14.1)	1.00
EuroScore	6 (4, 7)	6 (3, 8)	0.66
Single antiplatelet drug administration, *n* (%)	10 (10.1)	17 (17.2)	0.21
Dural antiplatelet drugs administration, *n* (%)	17 (17.2)	13 (13.1)	0.55
Anticoagulants administration only, *n* (%)	62 (62.6)	61 (61.6)	1.00
Antiplatelet drug and anticoagulants administration, *n* (%)	10 (10.1)	8 (8.1)	0.81
Reasons for catheterization, *n* (%)			
Tracheostomy	57 (57.6)	59 (59.6)	0.89
Hemodynamic monitoring	26 (26.3)	18 (18.2)	0.23
Catheter dysfunction	11 (11.1)	16 (16.2)	0.41
Suspicion of bloodstream infection	5 (5.1)	6 (6.1)	1.00
Patients on ECMO, *n* (%)	3 (3.0)	5 (5.1)	0.72
Patients on IABP, *n* (%)	3 (3.0)	5 (5.1)	0.72
Patients on RRT, *n* (%)	38 (38.4)	33 (33.3)	0.55
Duration of invasive mechanical ventilation, days	11 (6, 18)	11 (6, 19)	0.73
Median length of ICU stay, days	19 (12, 31)	18 (10, 26)	0.29
Median length of hospital stay, days	33 (23, 48)	29 (20, 43)	0.10
ICU mortality, *n* (%)	15 (15.2)	20 (20.2)	0.46
Hospital mortality, *n* (%)	16 (16.2)	22 (22.2)	0.37

Continuous data are presented as the mean (SD) or median (IQR). Categorical data are presented as counts (%)

*CABG* coronary artery bypass grafting, *ECMO* extracorporeal membrane oxygenation, *IABP* intra-aortic balloon pump, *RRT* renal replacement therapy

**Table 2:** Clinical parameters before venipuncture

At randomization	Proximal group (*n* = 99)	Distal group (*n* = 99)	*P* value
Heart rate (bpm)	90 (84, 100)	91 (84, 102)	0.80
Mean arterial pressure (mmHg)	75 (72, 79)	76 (72, 83)	0.47
Respiratory rate (cycle/min)	15 (15,1 5)	15 (15, 15)	0.63
CVP (mm Hg)	12 (10, 14)	12 (10, 14)	0.75
Need for mechanical ventilation (MV), *n* (%)	89 (90.0)	87 (87.9)	0.82
Side of catheterization (right), *n* (%)	83 (83.8)	79 (79.8)	0.58
Days after surgery, days	6 (4, 8)	6 (5, 8)	0.60
Tidal volume (mL/kg of PBW)	7.57 ± 1.32	7.73 ± 1.15	0.36
PEEP (cm H_2_O)	5 (5, 5)	5 (5, 5)	0.34
Vasopressor administration, *n* (%)	73 (73.7)	66 (66.7)	0.35
Inotropic administration, *n* (%)	66 (66.7)	63 (63.6)	0.77
Axillary vein depth (cm)	1.96 ± 0.51	2.45 ± 0.57	< 0.001
Axillary vein diameter (cm)	0.92 (0.77, 1.10)	0.75 (0.65, 0.87)	< 0.001
PT (s)	19.2 (14.5, 23.7)	19.4 (15.2, 26.6)	0.42
INR	1.73 (1.38, 2.12)	1.74 (1.39, 2.46)	0.37
Platelets (× 10^9^/L)	118 (86, 175)	128 (82, 184)	0.97

Continuous data are presented as the mean (SD) or median (IQR). Categorical data are presented as counts (%)

*PBW* predicted body weight, *PEEP* positive end-expiratory pressure, *CVP* central venous pressure, *PT* prothrombin time, *INR* international normalized ratio

All patients received oral antiplatelet drugs and/or anticoagulants for at least 3 days. The proportion of antiplatelet drugs and/or anticoagulants administered to the two groups was comparable. The median prothrombin time (PT), international normalized ratio (INR), and platelet count were similar between two groups (Table 2). AVC was indicated for tracheostomy in 116 (58.6%) patients, hemodynamic monitoring in 44 (22.2%), catheter dysfunction in 27 (13.6%), and suspicion of bloodstream infection in 11 (5.6%) patients. No patients underwent any corrective procedure for susceptibility to bleeding before venipuncture.

The PA group had higher first puncture success rate (75.8% vs. 51.5%, *p *< 0.001) and site success rate (93.9% vs. 83.8%, *p *= 0.04) than the DA group. However, the overall success rates between the PA and DA groups were similar (99.0% vs. 99.0%; *p *= 1.00). Moreover, the PA group had fewer average number of attempts (1.3 ± 0.7 vs. 1.7 ± 0.9; *p *= 0.002), less access time (20 s vs. 30 s, IQR: 15–28 and 19–42, respectively; *p *< 0.001), and less successful cannulation time (123 s vs. 142 s, IQR 112–136 and 133–156, respectively; *p *< 0.001) than the DA group.

The rates of total complications were comparable in both groups (9.1% vs. 11.1%, *p *= 0.81). None of the patients had major bleeding. The rates of minor bleeding were similar between the two groups (2.0% in the PA group and 5.1% in the DA group; *p *= 0.45). Furthermore, both groups showed no significant differences in other complications such as catheter misplacement, artery puncture, pneumothorax, and nerve injuries (Table 3).

**Table 3:** Outcome for proximal and distal approach groups using intention-to-treat (ITT) data sets

Success rates (ITT analysis)	Proximal group (*n* = 99)	Distal group (*n* = 99)	*p* value
Primary outcome			
First puncture success rate, *n* (%)	75 (75.8)	51 (51.5)	< 0.001
Secondary outcomes			
Approach success rate, *n* (%)	93 (93.9)	83 (83.8)	0.04
Overall success rate, *n* (%)	98 (99.0)	98 (99.0)	1.00
Average number of attempts	1.3 ± 0.7	1.7 ± 0.9	0.002
Access time (s)	20 (15, 28)	30 (19, 42)	< 0.001
Time to successful cannulation (s)	123 (112, 136)	142 (133, 156)	< 0.001
Total complications, *n* (%)	9 (9.1)	11 (11.1)	0.81
Major bleeding	0	0	–
Minor bleeding	2 (2.0)	5 (5.1)	0.45
Artery puncture	1 (1.0)	2 (2.0)	1.00
Pneumothorax	2 (2.0)	0	0.50
Nerve injuries	0	0	–
Catheter misplacements	5 (5.1)	7 (7.1)	0.77

Continuous data are presented as the mean (SD) or median (IQR). Categorical data are presented as counts (%)

## Discussion

This randomized controlled trial revealed that the PA had higher first puncture and site success rates than the DA for US-guided AVC in cardiac surgery patients susceptible to bleeding. However, the overall success rates between the PA and DA groups were similar. Moreover, the PA group had shorter access time and cannulation time than the DA group. The two groups, however, showed no differences in complications such as major bleeding, minor bleeding, arterial puncture, pneumothorax, nerve injuries, and catheter misplacement.

US-guided AVC has the advantages of SVC and causes fewer complications than the landmark method, which has become the ideal alternative choice [8, 21]. Two puncture approaches (proximal and distal) are usually used for AVC in clinical practice. To date, only one randomized trial has compared the two puncture approaches and reported that the success rates of both approaches were similar [15]. However, to our knowledge, no study has compared proximal and distal approaches for AVC in patients susceptible to bleeding.

For cardiac surgery patients, antiplatelet drugs and/or anticoagulants are usually used for preventing thrombosis; this is related to high bleeding risk [16]. Invasive procedures such as CVC may further put these patients at an additional risk of bleeding [17]. Limiting the number of venipuncture attempts could decrease the incidence of complications [22]. Therefore, we chose the first puncture success rate as the primary outcome of this trial.

Our results showed that the PA had higher first puncture and site success rates than the DA for US-guided AVC in cardiac surgery patients. The reasons for this finding can be summarized as follows. The anatomy of the infraclavicular axillary vein differs according to its position. As the direct continuation of the subclavian vein, the proximal axillary vein in the longitudinal axis view is straighter and thicker than the distal axillary vein [9]. The depth (the distance between skin surface and the anterior wall of the vein) of the proximal vein is smaller than that of the distal axillary vein [9]. Our results were consistent with previous reports. Compared with the distal approach, the vein diameter was larger and its depth was shorter in the proximal approach. The above anatomical features may contribute to a higher first puncture success rate for the proximal approach. All procedures in our study were performed by five clinicians with 3 to 18 months of experience in US-guided AVC. It is difficult to use a free-hand technique (nondominant hand holding the probe and dominant hand holding the needle) during the procedure for less experienced operators. The operators reported that the clavicle can serve as a support for the probe in the PA, whereas no obvious support was available in the DA. This may explain the higher first puncture success rate in the PA group.

Because cardiac surgery patients usually have chronic heart insufficiency, the central venous pressure (CVP) of our patients was higher than that of other ICU patients such as patients with sepsis [23, 24]. The CVP is often influenced by many factors such as cardiac function, position of the central catheter tip, mechanical ventilation, and use of vasodilating and vasoconstricting agents [25, 26]. Although CVP is unreliable for assessing volume status, higher CVP in cardiac surgery patients implies venous congestion to a certain extent [25, 27, 28], which favors the use of venipuncture. This can partly explain the difference in the success rate between the present study and other previous studies [12, 15, 29, 30].

The risk of bleeding depends on the experience of the operators, patients’ coagulation function, and whether US guidance is used [17]. Although the median PT and INR of the population before venipuncture in our study were higher than the normal range, many studies have reported that the coagulation test of PT or activated partial thromboplastin time (APTT) plays a limited role in predicting procedure-associated bleeding risk in critically ill patients [31, 32]. Viscoelastic coagulation tests such as thromboelastography may be an alternative method for predicting procedure-associated bleeding complications [33, 34]. In the present study, all patients received oral antiplatelet drugs or anticoagulants for at least 3 days, and none of the patients underwent any corrective procedure for susceptibility to bleeding before venipuncture. This implied an increased risk of bleeding. Recently, US has become a widely accepted guidance technique for safe and accurate CVC. The longitudinal axis/in-plane approach used for catheterization allowed real-time visualization of the entire needle (tip and shaft) during the procedure. It is pivotal to visualize the needle tip constantly during needle advancement, as it can decrease the risk of pleura and artery puncture [35]. The overall rates of artery puncture and pneumothorax in our study were lower than those noted in previous studies using landmark approaches [3, 8]. Moreover, there were no significant differences in complications including arterial puncture and pneumothorax between the PA group and the DA group. Hence, US-guided AVC can be considered as a safe method in patients with high risk of bleeding.

The present study had several limitations. First, long-term complications such as central line-associated bloodstream infection (CLA-BSI) and venous thrombosis were not recorded in this trial. Hence, no conclusion regarding these complications was derived from this study. Second, the short-axis approach was not included in the study. As longitudinal axis US-guided AVC has been safely and effectively used in our department for more than 5 years, we chose this procedure as the approach in this trial. Finally, although the term “axillary vein” was used in the distal approach, we acknowledge that in some cases, the main tributaries of the axillary vein (basilic vein) in the axilla were actually punctured.

## Conclusions

For cardiac surgery patients susceptible to bleeding, both proximal and distal approaches for US-guided AVC were considered as feasible and safe methods of central venous cannulation. In terms of the first puncture success rate and successful cannulation time, the PA was superior to the DA.

## Data Availability

All data generated or analyzed during this study are included in this published article.

## Abbreviations

CVC: Central venous catheterization
SVC: Subclavian vein catheterization
PA: Proximal approach
DA: Distal approach
CVP: Central venous pressure
PT: Prothrombin time
INR: International Normalized Ratio
APTT: Activated partial thromboplastin time
